# Cardiac adaptation in hibernating, free-ranging Scandinavian Brown Bears (*Ursus arctos*)

**DOI:** 10.1038/s41598-019-57126-y

**Published:** 2020-01-14

**Authors:** Peter Godsk Jørgensen, Alina Evans, Jonas Kindberg, Lisbeth Høier Olsen, Søren Galatius, Ole Fröbert

**Affiliations:** 1 University of Copenhagen, Department of Cardiology, Gentofte Hospital, Copenhagen, Denmark; 2 Department of Forestry and Wildlife Management, Inland Norway University of Applied Sciences, Campus Evenstad, Elverum, Norway; 3 Department of Wildlife, Fish and Environmental Studies, Faculty of Forest Sciences, Swedish University of Agricultural Sciences, Umeå, Sweden and Norwegian Institute for Nature Research, Trondheim, Norway; 4 Department of Veterinary and Animal Sciences, University of Copenhagen, Frederiksberg, Denmark; 5 University of Copenhagen, Department of Cardiology, Bispebjerg Hospital, Copenhagen, Denmark; 6 Örebro University Hospital, Faculty of Health, Department of Cardiology, Örebro, Sweden

**Keywords:** Cardiovascular diseases, Heart failure, Experimental models of disease

## Abstract

During six months of annual hibernation, the brown bear undergoes unique physiological changes to adapt to decreased metabolic rate. We compared cardiac structural and functional measures of hibernating and active bears using comprehensive echocardiography. We performed echocardiography on 13 subadult free-ranging, anaesthetised Scandinavian brown bears (*Ursus arctos*) during late hibernation and in early summer. Mean heart rate was 26 beats per minute (standard deviation (SD): 8) during hibernation vs 71 (SD: 14) during active state. All left ventricular (LV) systolic and diastolic measures were decreased during hibernation: mean ejection fraction: 44.2% (SD: 6.0) active state vs 34.0 (SD: 8.1) hibernation, P = 0.001; global longitudinal strain: −11.2% (SD: 2.0) vs −8.8 (SD: 3.3), P = 0.03; global longitudinal strain rate: −0.82 (SD: 0.15) vs −0.41 (SD: 0.18), P < 0.001; septal e’: 9.8 cm/s (SD: 1.8) vs 5.2 (SD: 2.7), P < 0.001. In general, measures of total myocardial motion (ejection fraction and global longitudinal strain) were decreased to a lesser extent than measures of myocardial velocities. In the hibernating brown bear, cardiac adaptation included decreased functional measures, primarily measures of myocardial velocities, but was not associated with cardiac atrophy. Understanding the mechanisms of these adaptations could provide pathophysiological insight of human pathological conditions such as heart failure.

## Introduction

To cope with the harsh environment during winter, the Scandinavian brown bear (*Ursus arctos*) has developed remarkable physiological adaptations. Each fall with a shortage of food and decreasing ambient temperature, the bear enters a 5–7 months hibernation period. During this time, the bear does not eat, drink, defecate or urinate and is only minimally physical active^1^. In addition, and contrary to most other hibernators, the Scandinavian brown bear is a shallow hibernator with a certain amount of alertness during the entire hibernation period and only a slight decrease in body temperature to approximately 33–34 °C^2^. Despite this extreme exposure the bear stays apparently free from any pathophysiological states associated with prolonged physical inactivity and avoids loss of muscle and bone mass^3–5^.

To conserve energy, the bear’s oxygen demand is reduced to approximately 25% of the active state^6^ and to optimise energy consumption the cardiovascular system adapts accordingly. Previous studies in American brown and black bears (*Ursus americanus*) have suggested that cardiac adaptations during hibernation are characterized by a marked decrease in cardiac output caused by profound bradycardia with extreme respiratory sinus arrhythmia and a preserved left ventricular (LV) ejection fraction. Hibernation is also characterized by a decrease in LV mass/volume ratio indicating some degree of cardiac remodelling to adapt to the altered hemodynamic state^7–9^. We have previously documented low flow hemodynamics in the hibernating Scandinavian brown bear by reporting reduced cardiac output and presence of spontaneous echo contrast^10^.

On the contrary, even a short period of immobility in humans can lead to a number of adverse effects including a reduction in muscle and bone mass, decubitus ulcers and thromboembolic events^11^. Cardiac implications of immobility documented in bed-rest studies^12^ and studies of astronauts returning from space^13^ have suggested cardiac atrophy to occur in response to immobility.

Over the past decade, several of echocardiographic modalities such as tissue Doppler imaging and 2D speckle tracking have been developed allowing detection of subtle changes in myocardial function. These methods allow assessment of LV myocardial tissue velocities and tissue deformation with high accuracy and have not been employed in a hibernating bear model previously. Additionally, previous studies examining cardiac structure and function have been conducted in hibernating bears that were hand-raised or in bears that were only examined during the hibernation period. Thus, we aimed to perform a comprehensive cardiac structural and functional evaluation in hibernating vs active free-ranging Scandinavian brown bears using tissue Doppler and 2D speckle tracking echocardiography and interpret the findings in a human context. Evidently, identification of the factors responsible for tissue preservation during hibernation has potential to impact human medicine in a variety of fields including cardiogenic shock, thromboembolism and space medicine.

## Methods

### Materials

We performed comprehensive echocardiography, including tissue Doppler imaging and 2D speckle tracking in free-ranging, subadult Scandinavian brown bears during the hibernating and active state in Dalarna, Sweden. All bears were marked with GPS collars and VHF transmitters as part of the Scandinavian Brown Bear Research Project (https://bearproject.info/) which allowed us to locate the bears in their dens during hibernation and in their habitat during the active state. Bears were immobilised in late February and again in June/early July from 2014 to 2018. During the winter captures, bears were located in their dens and anaesthetised with a mixture of medetomidine, zolazepam, tiletamine and ketamine. During summer captures, the same bears were darted from a helicopter with a mixture of medetomidine, zolazepam and tiletamine at 2–4 times the winter dose to account for the metabolic rate during active state^14^. Both winter and summer, ketamine could be administered if the effect of anaesthesia was decreasing. Doses administered and weight of each of the bears is shown in Supplemental Table 1. The study was approved by the Swedish Ethical Committee on Animal Research (C268/12 and C3/16), and the procedures were performed in compliance with Swedish laws and regulations.

### Echocardiography

**Figure 1:**
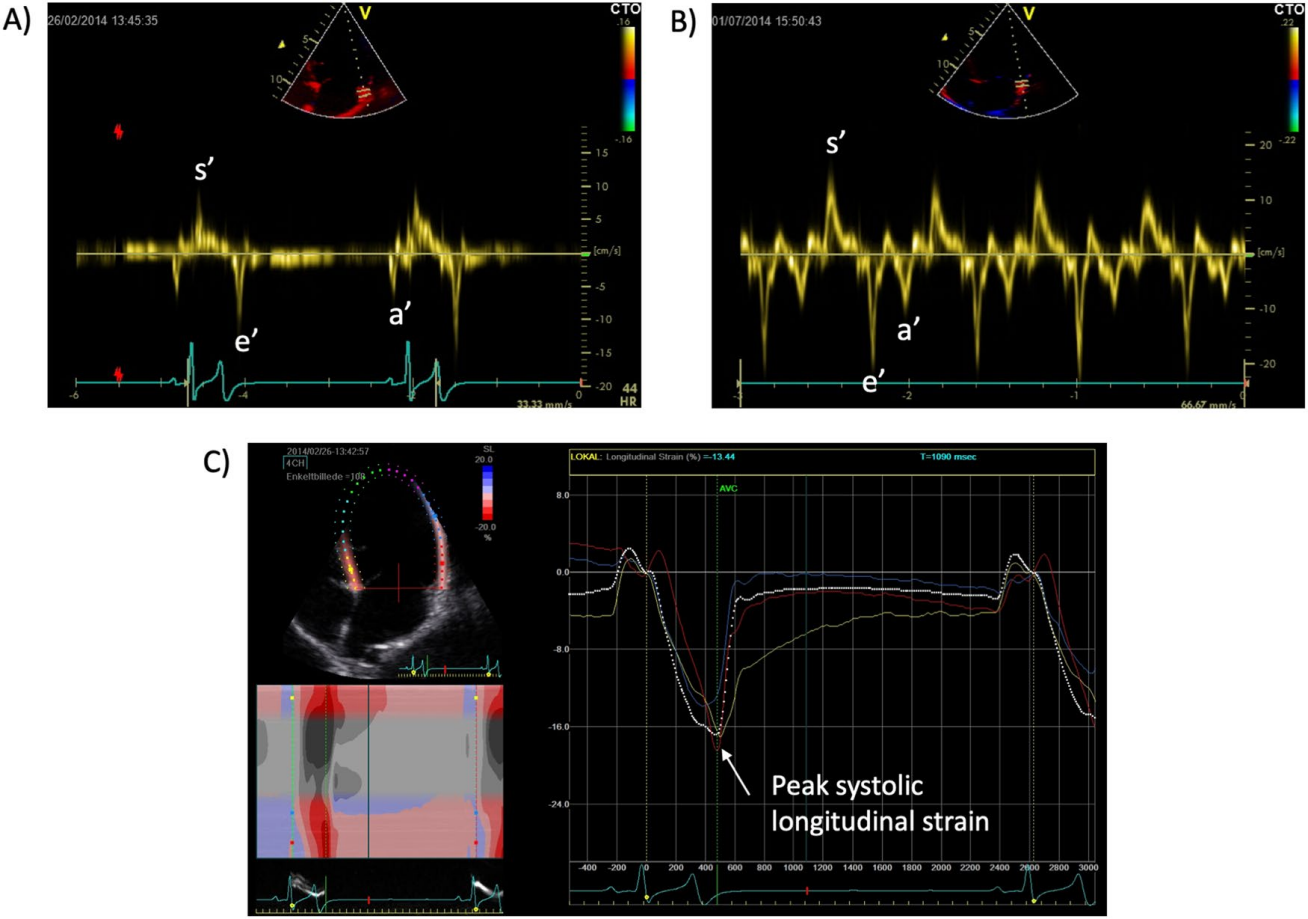
Examples of pulsed wave tissue Doppler echocardiographic and 2D-speckle tracking measurements. S’, e’ and a’ during hibernation (**A**) and active state (**B**) and of 2D-speckle tracking strain measurements with an arrow pointing at peak systolic strain (**C**). Images were exported from EchoPAC 113.1.5.6, GE Vingmed Ultrasound AS, Strandpromenaden 45, 3191 Horten, Norway. URL: https://www.gehealthcare.com/.

We performed echocardiography in the field on bears in a left lateral recumbency position. We used a General Electrics Vivid i (GE healthcare, Horton, Norway) with 5S-RS and 3S-RS probes to obtain 2D images, M-mode, pulsed, continuous wave and tissue Doppler echocardiography. We used GE EchoPAC software (EchoPAC 113.1.5.6, GE Vingmed Ultrasound AS, Strandpromenaden 45, 3191 Horten, Norway) to perform post-processing of the echocardiograms. Three apical views (4-chamber, 2-chamber and apical long-axis view) were recorded and, where possible, a parasternal long-axis view was also obtained. Following the European Association of Cardiovascular Imaging guidelines, LV mass was approximated with the Cube formula that has been validated in humans (LV mass = 0.8 × {1.04[(LV internal diameter + posterior wall thickness + septal wall thickness)^3^ − (LV internal diameter)^3^]} + 0.6 g)^15^. LV dimensions were measured in end-diastole in the parasternal long-axis view and indexed according to estimated body surface^16^ to account for differences related to the growth of the subadult animals. 2D-speckle tracking strain and strain rate were measured in the apical 4-, 2- and long axis view in a total of 18 segments. The region of interest was traced by a semi-automated process and adjusted manually to cover the entire LV wall after visual inspection. Segments that were not traced by the software were excluded from the analyses. The mean values of mid-ventricular global longitudinal strain and strain rate from the three projections reported by the software were used in this study. An example is shown in Fig. 1C. Mitral valve inflow velocities, early (E) and atrial (A) velocities and deceleration time were measured with the sample volume placed between the tips of the mitral valve leaflets. Peak early (e’), atrial (a’) and systolic (s’) myocardial velocities were measured with pulsed-wave tissue Doppler with the sample volume placed in the septal and lateral mitral annulus as demonstrated in Fig. 1A,B. Isovolumetric relaxation time was calculated from these curves as the time between end of systolic to onset of early diastolic myocardial velocity wave and reported as the mean of the septal and lateral values. Left atrial end volumes and emptying/ejection fraction were calculated using Simpson’s biplane method from apical 2- and 4-chamber views^15^. Right ventricular fractional area change was calculated as (end-diastolic area – end-systolic area)/end-diastolic area, measured in a modified apical 4-chamber view with a focus on the right ventricle. Tricuspid annular plane systolic excursion (TAPSE) was also obtained in this view with use of M-mode. All measurements were obtained in all bears except right ventricular area measurements that were not analyzable in one bear. Heart rate was the mean of 3 measurements performed during the 2D speckle tracking analyses. An experienced echocardiographer (PGJ) performed all echocardiographies and post-processing.

### Statistics

The values are presented as mean ± standard deviation (SD), and differences between hibernating and active state were compared using a paired t-test. P-values less than 0.05 were considered statistically significant. The calculations were made using the statistical software package ‘R’, version 3.4.3 (64 bit) (R Project for Statistical Computing, http://www.R-project.org).

## Results

A total of 13 bears, 4 males and 9 females, were studied during the hibernating and active state. Two of the bears were studied two consecutive years and included in the analyses separately. All bears were subadults, three were 3 years old, and nine were 2 years old. Mean weight was 45.6 kg (SD: 9.3) in the active state versus 40.5 kg (SD: 10.9) during hibernation (p = 0.02). Heart rate was considerably lower during hibernation, 26 beats per minute (SD: 8) vs 71 (SD: 14) during active state (p < 0.001).

**Figure 2:**
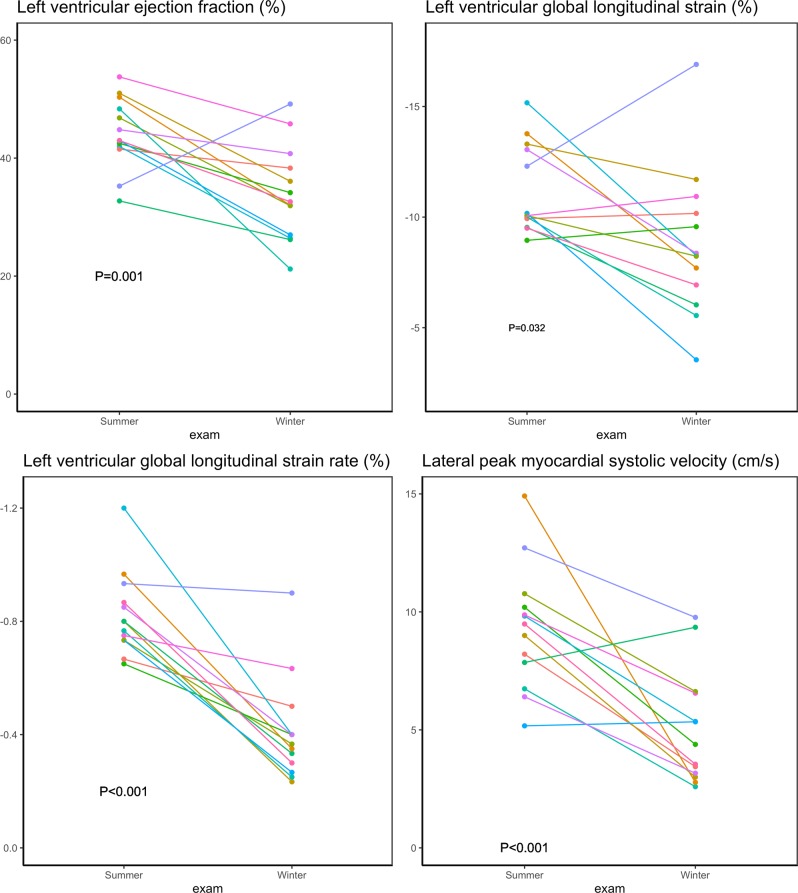
Systolic functional measures in active vs hibernating Scandinavian Brown Bear.

**Figure 3:**
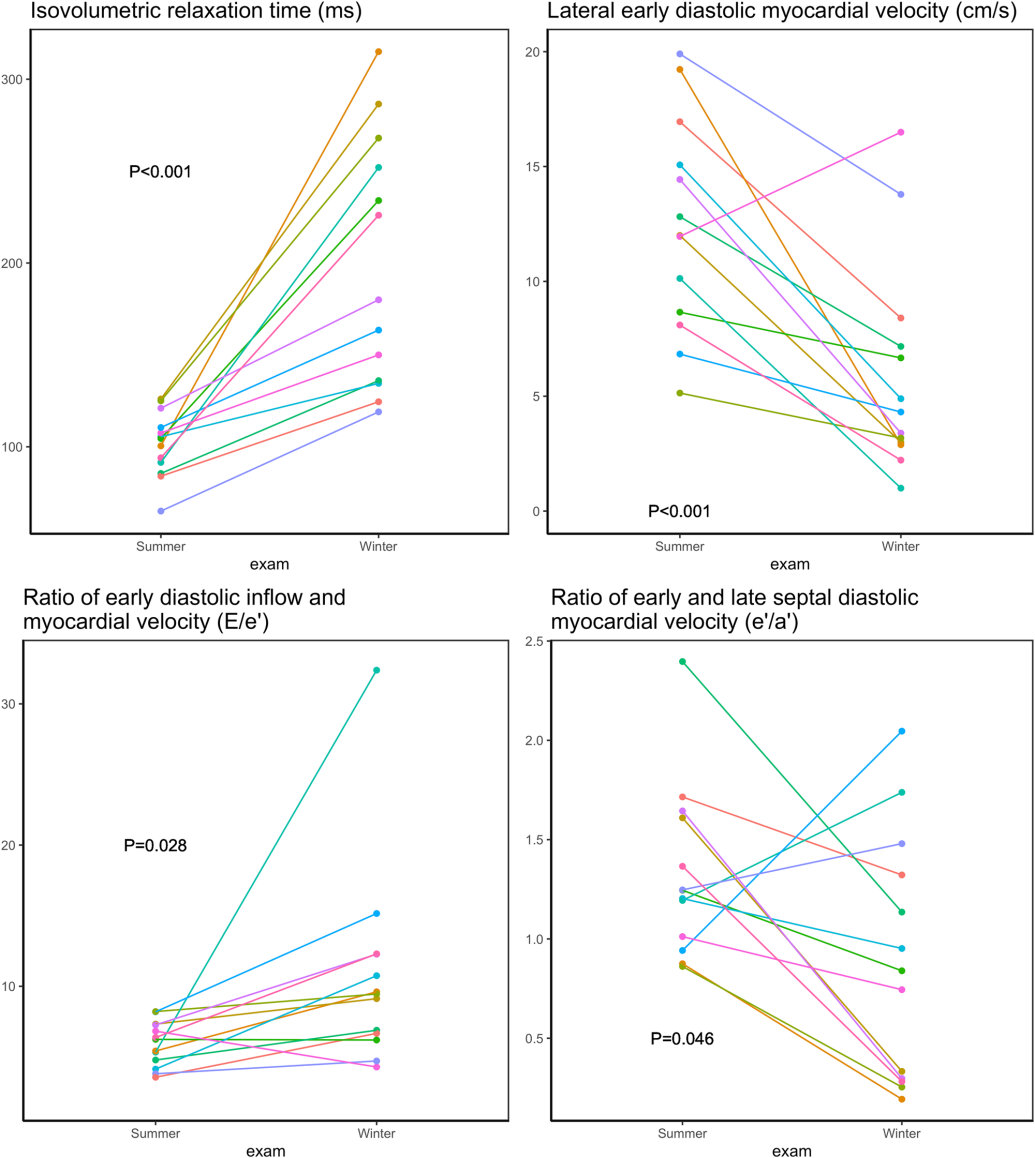
Diastolic functional measures in active vs hibernating Scandinavian Brown Bear.

**Figure 4:**
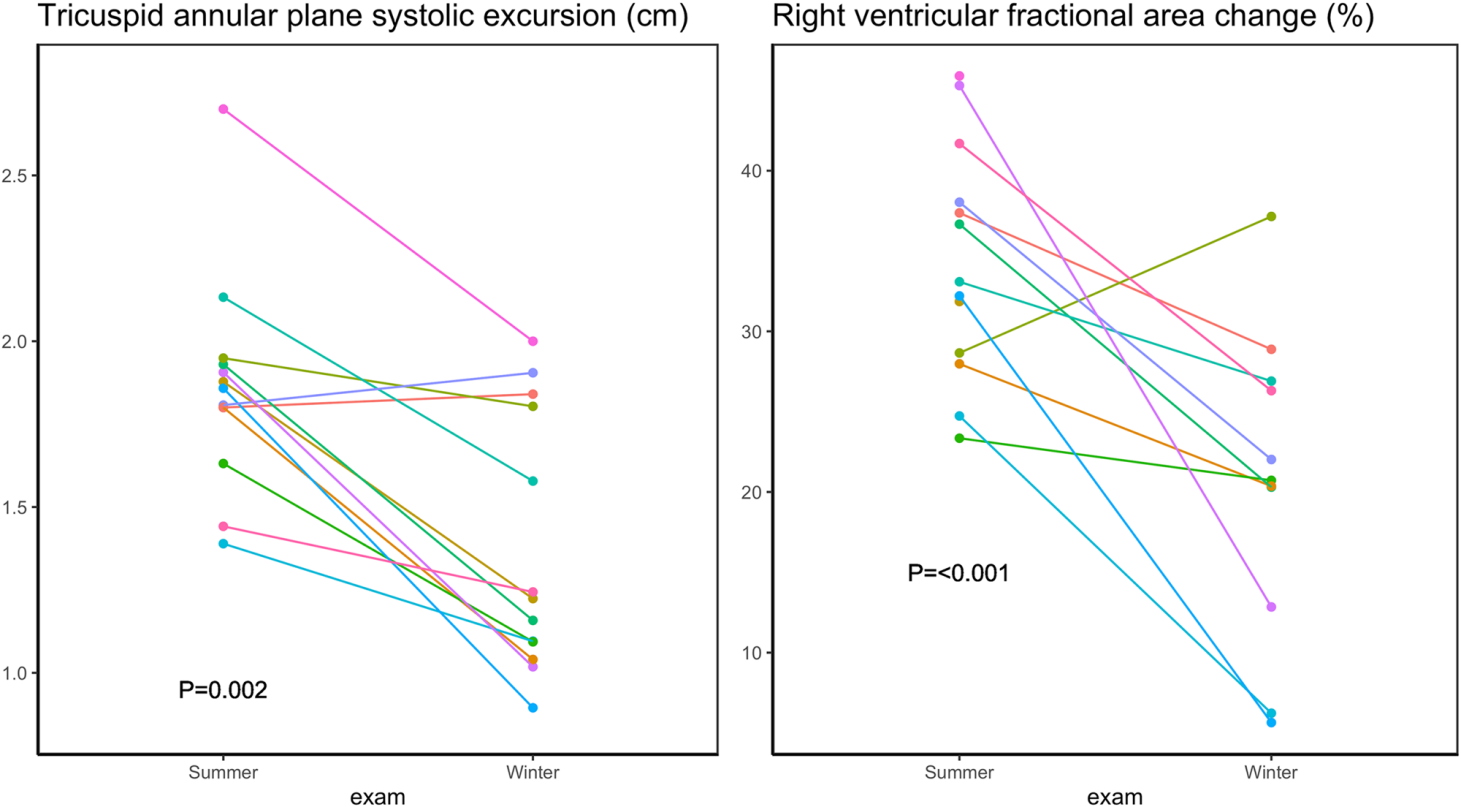
Right ventricular functional measures in active vs hibernating Scandinavian Brown Bear.

The echocardiographic measurements are presented in Table 1. None of the structural measurements were significantly changed during hibernating compared to an active state. Thus, neither LV mass, myocardial wall thicknesses nor volumes were altered (p > 0.05 for all). In contrary, the majority of the functional measurements were reduced during hibernation. Regarding systolic measurements, LV ejection fraction, global longitudinal strain and strain rate and peak systolic velocities were reduced during hibernation, Fig. 2. This pattern was, however, more pronounced regarding the myocardial velocity measurements. While the measures of total cardiac *motion* were only modestly affected with a 23% relative reduction in LV ejection fraction and 21% reduction in global longitudinal strain, the measures of myocardial *velocities* were reduced to a larger extent - global longitudinal strain rate by 50% and septal and laterals’ by 39% and 45%, respectively. In contrast to the LV measures, neither left atrial structure nor function (except lateral a’) were significantly altered during the active state. LV diastolic measurements, Table 1 and Fig. 3, were all significantly lower during hibernation compared to active state except mitral valve inflow deceleration time, E/A ratio and lateral e’/a’. The E/e’ seemed to be highly affected by one observation, Fig. 3. By excluding this bear, the increase in E/e’ during hibernation is still significant, P = 0.02. This pattern of preserved structure and reduced function was also found in right ventricular measurements, where unaltered systolic and diastolic areas were accompanied by significantly decreased functional measures expressed as tricuspid annular plane systolic excursion (TAPSE) and fractional area change, Fig. 4.

**Table 1:** Echocardiographic findings in active vs hibernating Scandinavian Brown Bear.

	Active state Mean (sd)	Hibernating state Mean (sd)	p-value
***Left ventricular structure***			
Left ventricular mass index (g/m^2^)	171 (24)	151 (28)	0.07
Septum thickness index (cm/m^2^)	0.91 (0.16)	0.83 (0.12)	0.19
Diastolic internal diameter (cm/m^2^)	4.1 (0.4)	4.5 (0.7)	0.14
Posterior wall thickness (cm/m^2^)	0.81 (0.13)	0.77 (0.11)	0.40
Left ventricular end diastolic volume (ml/m^2^)	105 (18)	94 (4)	0.12
Left ventricular end systolic volume (ml/m^2^)	58 (12)	62 (12)	0.43
***Left ventricular systolic function***			
Left ventricular ejection fraction (%)	44.2 (6.0)	34.0 (8.1)	0.001
Global longitudinal strain (%)	−11.2 (2.0)	−8.8 (3.3)	0.032
Global longitudinal strain rate (s^−1^)	−0.82 (0.15)	−0.41 (0.18)	<0.001
Septal s’ (cm/s)	7.5 (1.1)	4.6 (1.9)	<0.001
Lateral s’ (cm/s)	9.3 (2.6)	5.1(2.4)	<0.001
***Left ventricular diastolic function***			
Isovolumetric relaxation time (ms)	102 (18)	199 (68)	<0.001
Mitral valve inflow deceleration time (ms)	96 (31)	123 (76)	0.23
A velocity (m/s)	0.48 (0.17)	0.33 (0.08)	0.009
E velocity (m/s)	0.69 (0.20)	0.45 (0.16)	0.003
E/A ratio	1.6 (0.6)	1.4 (0.6)	0.58
Lateral e’ (cm/s)	12.4 (4.6)	6.0 (4.6)	0.002
septal e’ (cm/s)	9.8 (1.8)	5.2 (2.7)	<0.001
e’/a’ lateral	2.0 (0.9)	1.2 (0.9)	0.053
e’/a’ septal	1.3 (0.4)	0.9 (0.6)	0.046
E/e'	6.0 (1.6)	10.8 (7.2)	0.028
***Left atrial structure and function***			
Left atrial end systolic volume index (ml/m^2^)	25 (10)	23 (5)	0.41
Left atrial end diastolic volume index (ml/m^2^)	41 (11)	37 (7)	0.34
Left atrial emptying fraction (%)	38 (18)	38 (14)	0.90
Septal a’ (cm/s)	7.7 (1.3)	7.3 (2.6)	0.61
Lateral a’ (cm/s)	6.7 (1.8)	4.9 (1.5)	0.010
***Right ventricular function***			
TAPSE (cm)	1.9 (0.3)	1.4 (0.4)	0.002
*Systolic area (cm^2^)	8.3 (2.3)	8.5 (1.8)	0.81
*Diastolic area (cm^2^)	12.5 (3.1)	10.7 (2.3)	0.13
*Fractional area change (%)	34.3 (7.3)	20.5 (9.1)	<0.001
• Indicates measures only available in 12 bears.

## Discussion

We have examined cardiac adaptations to the annual hibernation in free-ranging Scandinavian brown bears using contemporary, comprehensive echocardiography. While there was no evidence of structural adaptation such as atrophy during hibernation, we found that both left and right ventricular systolic and LV diastolic function were reduced during hibernation. Interestingly, using advanced measures of cardiac deformation and tissue velocities, we found that the relative decrease in function was largest for velocity measures such as global longitudinal strain rate, s’ and e’, compared to the volumetric and maximum deformation measures. This indicates that cardiac adaptation affects tissue velocities more than the actual magnitude of contraction. Of note, we did not find any difference in atrial size nor function (except lateral a’, which was lower during hibernation) and only a borderline change in early and late diastolic relative contribution to LV filling expressed as E/A ratio, e’/a’.

To the best of our knowledge this study is the first to demonstrate considerably reduced LV myocardial velocities using new, sensitive measures, of myocardial function, i. e. tissue Doppler imaging and 2D speckle tracking imaging in hibernating bears. In humans, heart failure – defined as a chronic condition in which the heart’s ability to pump blood is unable to meet the tissue’s oxygen demands at normal filling pressures – is also characterised by decreased myocardial velocities that have consistently shown to be associated with poor prognosis^17–21^. Though the bear shares similarities to patients with heart failure, including reduced ejection fraction and myocardial velocities, these are not associated with any pathophysiological symptoms or prognosis in hibernating bears. In heart failure, decreased left and right ventricular function is associated with cardiac remodelling as a compensatory mechanism. In the early stages, heart failure with preserved ejection fraction is associated with increased filling pressures leading to pressure increase in and dilation of the left atrium. At a later stage in heart failure with reduced ejection fraction, to compensate for decreased stroke volume, LV remodelling including increasing volumes and wall thickness ensure to maintain adequate stroke volume and cardiac output^22^. According to our results, the bear’s heart does not remodel as a compensatory mechanism to decreased myocardial function indicating perfect adaptation during hibernating. We believe that the reduced function is not accompanied by an increase in filling pressures because the cardiovascular system during hibernation is a low flow system as we have previously described^10^.

The fact that the bear is able to modulate contractile forces during hibernation underlines the prospects of the hibernating bear heart as a useful model of understanding heart diseases in humans. Revealing the mechanisms of these modulations could have potentially considerable benefits in human medicine. In our study, the myocardial velocities were more decreased compared to volumetric and deformational measures. In the cardiac myocyte, the force generated is dependent on intracellular Ca^2+^ released from the sarcoplasmic reticulum during contraction and sensitivity of myosin filaments to Ca^2+^ ^23^. Also, previous studies have suggested that body temperatures below 30 °C are required to effectively alter Ca^2+^ dynamics^24,25^ and, therefore, temperature alone is unlikely to be responsible for the cardiac adaptations found as the hibernating brown bear keeps a temperature at approximately 33 °C during hibernation^2^. As a consequence, our study indicates, that handling of Ca^2+^ and the sensitivity to Ca^2+^ in contractile proteins are potentially involved in the myocardial adaptations in the hibernating brown bear.

Our finding of a preserved cardiac structure is in accordance with previous studies on cardiac structure in hibernating bears. In 2003 and 2008 Nelson *et al*. conducted research on captive, hibernating brown bears. In the first study, they found no difference in septal thickness^8^, whereas in the second, bears showed reduced septal thickness^26^. Both studies, however, reported unaltered LV end-diastolic diameters during hibernation. Laske *et al*. studied the structural changes in black bears from early through late hibernation period and found no differences in LV wall thicknesses^7^.

In our study, all systolic measures were reduced during hibernation, including measures of myocardial velocities such as global longitudinal strain, strain rate and s’ that were relatively more reduced than measures of total cardiac motion such as LV ejection fraction and global longitudinal strain. Our results contrast findings of Nelson *et al*., who initially also reported a decreased LV ejection fraction in anaesthetised captive brown bears^8^, but later reported preserved ejection fraction in unanaesthetised captive brown bears^9^. An apparent reason for this discrepancy could be the use of anaesthesia in our bears. In Nelson’s study of anesthetised bears, a cocktail of tiletamine and zolazepam was administered to induce and isoflurane was used to sustain anesthesia during the examination. The isolated effect of isoflurane on the bear heart is unknown, but in rabbits isoflurane lowers blood pressure and cardiac function^27^. In humans, on the other hand, isoflurane only has limited effect on cardiac function^28^, though this might be age dependent^29^. Thus, the reduced ejection fraction in anesthetised bears in Nelson’s early study might be caused by the use of isoflurane but this remains speculative. Another reason for the discrepancy could also be due to differences in image acquisition and calculations of LV ejection fraction. In our study, we used Simpson’s biplane method which is the currently recommended method in clinical practice in humans^15^ and Nelson *et al*. used area length method from the parasternal long axis view (Nelson, personal communication). The atrial function was unaltered during hibernation in our study. This result is in discrepancy with the findings of Nelson *et al*. who described decreased atrial function – by both 2D and strain echocardiography - during hibernation and ascribed this as a consequence/adaptation to increased LV stiffness interpreted as increased isovolumetric relaxation time^9,30^. Isovolumetric relaxation time was also dramatically increased during hibernation in our study. However, based partly on our findings of altered myocardial velocities, we believe that this is mainly caused by other factors than increased myocardial stiffness: isovolumetric relaxation time is a marker of decreased diastolic function and is affected in a number of diseases, but is also dependent of end-systolic left atrial pressure and shortens with increasing left atrial pressure and vice versa^31^. Beyond this, LV relaxation rate is determined by reuptake of Ca^2+^ in the sarcoplasmic reticulum of the myocyte. This process occurs through the action of sarco(endo)plasmic reticulum Ca^2+^ ATPase which is highly sensitive to adrenergic stimuli^32^. Thus, with respect to our previous finding of low cardiac output in hibernating brown bears, we believe that the prolonged isovolumetric relaxation time is caused by low end-systolic left atrial pressure as well as a presumed minimal adrenergic state in bears during hibernation.

We have demonstrated profound changes in cardiac function during hibernation in Scandinavian brown bears. The characteristics of myocardial metabolism during the hibernating state is unknown. Free fatty acid oxidation is responsible for approximately 70% of myocardial energy expenditure in the healthy human heart and up to a 70% decrease in free fatty acid oxidation has been observed in the failing heart accompanied by decreased substrate flexibility^33,34^. In the search for innovative treatments targeting fatty acid metabolism in heart failure, knowledge of adaptations in metabolism during hibernation might prove useful^35^. Latest could be investigated using injected tracers that are examined either directly from blood samples from cardiac catheterization^33^ or by positron emission tomography^36^. At present, Ca^2+^ metabolism is already an established target in heart failure therapy in humans with the agent *levosimendan* which is a Ca^2+^-sensitizer acting through binding to the cardiac troponin C molecule – a modulator of force of contraction in the cardiomyocyte^37^. However, further knowledge of the bear’s possible ability to effectively control Ca^2+^ transfer? is essential for providing insights into the mechanisms required to effectively control this process.

A strength of this study is that we studied free-ranging Scandinavian brown bears in their natural habitat, including natural denning behaviour and denning physiology. Also, we examined the highest number of hibernating and active bears reported to date using echocardiography. A significant limitation is that we used bears anaesthetised with agents possibly affecting cardiac functional measures. This may explain the slightly depressed LV function during active state although no previous studies have established average values of LV ejection fraction using the gold standard Simpson’s biplane method and global longitudinal strain in unanaesthetised active brown bears.

Ketamine was only used on a routine basis during winter captures and since ketamine is believed to have centrally mediated sympathomimetic effects^38^, the use of ketamine would theoretically cause changes opposite to those observed in this study. Additionally, during anaesthesia, we used doses that were titrated based on careful monitoring according to the depth of anaesthesia during both summer and winter captures. Moreover, we found no consistent correlation between dose of the anaesthetic agents and the cardiac measures (data not shown). On the other hand, anaesthesia allowed us to examine the bear heart from the apical position by applying firm pressure to the thoracic wall and slightly displace the sternum which is unlikely to be possible in wake bears. By doing this, we could obtain reliable Doppler measurements (both spectral and tissue Doppler) that are otherwise angle sensitive.

## Conclusion

We used comprehensive echocardiography to characterise cardiac adaptations in hibernating brown bears. While we found no evidence of cardiac atrophy during hibernation, we demonstrated a decrease in functional measures. The measurements of cardiac velocities changed the most, which may indicate changes to Ca^2+^ transfer handling during hibernation. Identification of factors responsible for the remission of decreased cardiac functional measures could have implications for the management of human patients with a number of conditions including heart failure.

## Supplementary information

Supporting Information.

Supporting Information 2.

## Data Availability

The data will be made available upon reasonable request.
