# Supplementary material for: Cardiac adaptation in hibernating, free-ranging Scandinavian Brown Bears (Ursus arctos)
Source: Sci Rep. 2020 Jan 14;10:247. doi: 10.1038/s41598-019-57126-y (PMC6959366; doi:10.1038/s41598-019-57126-y)
Supplement: Supplementary file 1 — Supporting Information. [file 41598_2019_57126_MOESM1_ESM.docx]

Cardiac adaptation in hibernating, free-ranging Scandinavian Brown Bears (*Ursus arctos*)

Peter Godsk Jørgensen^1^, Alina Evans^2^, Jonas Kindberg^3^, Lisbeth Høier Olsen^4^, Søren Galatius^5^ and Ole Fröbert^6^

1. University of Copenhagen, Department of Cardiology, Gentofte Hospital, Copenhagen, Denmark, 2. Department of Forestry and Wildlife Management, Inland Norway University of Applied Sciences, Campus Evenstad, Elverum, Norway, 3. Department of Wildlife, Fish and Environmental Studies, Faculty of Forest Sciences, Swedish University of Agricultural Sciences, Umeå, Sweden and Norwegian Institute for Nature Research, Trondheim, Norway, 4. Department of Veterinary and Animal Sciences, University of Copenhagen, Frederiksberg, Denmark, 5. University of Copenhagen, Department of Cardiology, Bispebjerg Hospital, Copenhagen, Denmark and 6. Örebro University Hospital, Faculty of Health, Department of Cardiology, Örebro, Sweden

*Corresponding author:*

Peter Godsk Jørgensen, Department of Cardiology, Gentofte University Hospital, Gentofte Hospitalsvej 1, 2900 Hellerup, Copenhagen, Denmark, [petergodsk@gmail.com](mailto:petergodsk@gmail.com), tel: +45 22 48 51 75, fax: +45 39 77 73 81

Supplemental table 1. Body weight and doses administered during anaesthesia.

| **Bear** | **State** | **Body weight (kg)** | **Medetomidine (mg)** | **Zolatil (mg)** | **Ketamine (mg)** |
| --- | --- | --- | --- | --- | --- |
| 1 | Active state | 43,00 | 2,50 | 125,00 | 200,00 |
| 2 | Active state | 33,00 | 2,50 | 125,00 | 300,00 |
| 3 | Active state | 49,00 | 2,50 | 125,00 | 0,00 |
| 4 | Active state | 42,00 | 2,50 | 125,00 | 70,00 |
| 5 | Active state | 40,00 | 2,50 | 125,00 | 100,00 |
| 6 | Active state | 45,00 | 5,00 | 250,00 | 100,00 |
| 7 | Active state | 68,00 | 5,00 | 250,00 | 0,00 |
| 8 | Active state | 53,40 | 2,50 | 125,00 | 30,00 |
| 9 | Active state | 48,00 | 5,00 | 250,00 | 0,00 |
| 10 | Active state | 42,40 | 5,00 | 250,00 | 0,00 |
| 11 | Active state | 55,00 | 5,00 | 250,00 | 100,00 |
| 13 | Active state | 37,00 | 2,50 | 125,00 | 70,00 |
| 14 | Active state | 37,00 | 2,50 | 125,00 | 290,00 |
| 1 | Hibernation | 36,00 | 0,65 | 62,50 | 50,00 |
| 2 | Hibernation | 31,50 | 0,65 | 62,50 | 37,50 |
| 3 | Hibernation | 37,00 | 0,65 | 62,50 | 77,50 |
| 4 | Hibernation | 28,00 | 0,65 | 62,50 | 37,50 |
| 5 | Hibernation | 33,00 | 0,65 | 62,50 | 77,50 |
| 6 | Hibernation | 45,00 | 1,25 | 125,00 | 175,00 |
| 7 | Hibernation | 72,00 | 1,25 | 125,00 | 75,00 |
| 8 | Hibernation | 39,50 | 0,65 | 62,50 | 37,50 |
| 9 | Hibernation | 43,50 | 0,65 | 62,50 | 37,50 |
| 10 | Hibernation | 33,50 | 0,65 | 62,50 | 75,00 |
| 11 | Hibernation | 45,00 | 0,65 | 62,50 | 167,50 |
| 13 | Hibernation | 42,50 | 0,65 | 62,50 | 75,00 |
| 14 | Hibernation | 40,50 | 0,65 | 62,50 | 37,50 |
