# Supplementary material for: Cardiac adaptation in hibernating, free-ranging Scandinavian Brown Bears (Ursus arctos)
Source: Sci Rep. 2020 Jan 14;10:247. doi: 10.1038/s41598-019-57126-y (PMC6959366; doi:10.1038/s41598-019-57126-y)
Supplement: Supplementary file 2 — Supporting Information 2. [file 41598_2019_57126_MOESM2_ESM.pdf]

Cardiac adaptation in hibernating, free-ranging Scandinavian Brown Bears (*Ursus arctos*)

Peter Godsk Jørgensen[1], Alina Evans[2], Jonas Kindberg[3], Lisbeth Høier Olsen[4], Søren Galatius[5] and Ole Fröbert[6]

1. University of Copenhagen, Department of Cardiology, Gentofte Hospital, Copenhagen, Denmark, 2. Department of Forestry and Wildlife Management, Inland Norway University of Applied Sciences, Campus Evenstad, Elverum, Norway, 3. Department of Wildlife, Fish and Environmental Studies, Faculty of Forest Sciences, Swedish University of Agricultural Sciences, Umeå, Sweden and Norwegian Institute for Nature Research, Trondheim, Norway, 4. Department of Veterinary and Animal Sciences, University of Copenhagen, Frederiksberg, Denmark, 5. University of Copenhagen, Department of Cardiology, Bispebjerg Hospital, Copenhagen, Denmark and 6. Örebro University Hospital, Faculty of Health, Department of Cardiology, Örebro, Sweden

Corresponding author:

Peter Godsk Jørgensen, Department of Cardiology, Gentofte University Hospital, Gentofte Hospitalsvej 1, 2900 Hellerup, Copenhagen, Denmark, petergodsk@gmail.com, tel: +45 22 48 51 75, fax: +45 39 77 73 81

Supplemental table 1. Body weight and doses administered during anaesthesia.

Bear	State	Body weight (kg)	Medetomidine (mg)	Zolatil (mg)	Ketamine (mg)
1	Active state	43,00	2,50	125,00	200,00
2	Active state	33,00	2,50	125,00	300,00
3	Active state	49,00	2,50	125,00	0,00
4	Active state	42,00	2,50	125,00	70,00
5	Active state	40,00	2,50	125,00	100,00
6	Active state	45,00	5,00	250,00	100,00
7	Active state	68,00	5,00	250,00	0,00
8	Active state	53,40	2,50	125,00	30,00
9	Active state	48,00	5,00	250,00	0,00
10	Active state	42,40	5,00	250,00	0,00
11	Active state	55,00	5,00	250,00	100,00
13	Active state	37,00	2,50	125,00	70,00
14	Active state	37,00	2,50	125,00	290,00
1	Hibernation	36,00	0,65	62,50	50,00
2	Hibernation	31,50	0,65	62,50	37,50
3	Hibernation	37,00	0,65	62,50	77,50
4	Hibernation	28,00	0,65	62,50	37,50
5	Hibernation	33,00	0,65	62,50	77,50
6	Hibernation	45,00	1,25	125,00	175,00
7	Hibernation	72,00	1,25	125,00	75,00
8	Hibernation	39,50	0,65	62,50	37,50
9	Hibernation	43,50	0,65	62,50	37,50
10	Hibernation	33,50	0,65	62,50	75,00
11	Hibernation	45,00	0,65	62,50	167,50
13	Hibernation	42,50	0,65	62,50	75,00
14	Hibernation	40,50	0,65	62,50	37,50
